# Crop host signatures reflected by co-association patterns of keystone *Bacteria* in the rhizosphere microbiota

**DOI:** 10.1186/s40793-021-00387-w

**Published:** 2021-10-12

**Authors:** Simon Lewin, Davide Francioli, Andreas Ulrich, Steffen Kolb

**Affiliations:** 1 Microbial Biogeochemistry, Research Area Landscape Functioning, Leibniz Centre for Agricultural Landscape Research e.V. (ZALF), Müncheberg, Germany; 2 Thaer Institute, Faculty of Life Sciences, Humboldt University of Berlin, Berlin, Germany

**Keywords:** 16S rRNA, Amplicon sequencing, Barley, Co-occurrence network, *LEfSe*, Oilseed rape, RNA, Rye, Wheat

## Abstract

**Background:**

The native crop bacterial microbiota of the rhizosphere is envisioned to be engineered for sustainable agriculture. This requires the identification of keystone rhizosphere *Bacteria* and an understanding on how these govern crop-specific microbiome assembly from soils. We identified the metabolically active bacterial microbiota (SSU RNA) inhabiting two compartments of the rhizosphere of wheat (*Triticum aestivum* L.), barley (*Hordeum vulgare* L.), rye (*Secale cereale*), and oilseed rape (*Brassica napus* L.) at different growth stages.

**Results:**

Based on metabarcoding analysis the bacterial microbiota was shaped by the two rhizosphere compartments, i.e. close and distant. Thereby implying a different spatial extent of bacterial microbiota acquirement by the cereals species versus oilseed rape. We derived core microbiota of each crop species. *Massilia* (barley and wheat) and unclassified Chloroflexi of group ‘KD4-96’ (oilseed rape) were identified as keystone *Bacteria* by combining *LEfSe* biomarker and network analyses. Subsequently, differential associations between networks of each crop species’ core microbiota revealed host plant-specific interconnections for specific genera, such as the unclassified *Tepidisphaeraceae* ‘WD2101 soil group’.

**Conclusions:**

Our results provide keystone rhizosphere *Bacteria* derived from for crop hosts and revealed that cohort subnetworks and differential associations elucidated host species effect that was not evident from differential abundance of single bacterial genera enriched or unique to a specific plant host. Thus, we underline the importance of co-occurrence patterns within the rhizosphere microbiota that emerge in crop-specific microbiomes, which will be essential to modify native crop microbiomes for future agriculture and to develop effective bio-fertilizers.

**Supplementary Information:**

The online version contains supplementary material available at 10.1186/s40793-021-00387-w.

## Background

Crop plants are colonized by a microbiota. This symbiotic association constitutes a holobiont that responds jointly to environmental factors [1–3]. A systemic understanding of the crop bacterial microbiota and utilization of its plant-beneficial effects may allow for plant productions systems that will be more resilient to climate change and may address sustainability concerns [4, 5]. Current crop cultivars require nutrient fertilization and further agrochemicals to allow for stable and sufficient yields. However, agrochemical inputs lead eventually to an increasing environmental pollution and biodiversity losses [6, 7].

Specific interactions of crop rhizosphere microorganisms modulate and improve plant resilience against environmental stressors by (i) mobilization of plant nutrients, (ii) growth promotion through plant hormone synthesis, (iii) systemic or indirectly induced pathogenic resistance, and (iv) antagonistic effects on pathogens [8, 9]. These specific interactions are realized by the diverse microbial species of the same crop microbiota. An important factor that supports microbial growth in the rhizosphere making it to a microbial biomass and activity hotspot adjacent to the soil is the release of rhizodeposits. These foster niche differentiation nearby the root [10] and fuel microbial growth [11, 12]

Differences in physicochemical soil characteristics have the strongest impact on the rhizosphere bacterial microbiota composition. They have been proven for plant species such as *Arabidopsis thaliana* [13, 14], *Hordeum vulgare* L. (barley) [15] and *Triticum aestivum* L. (wheat) [16, 17] but also for complex plant communities in grasslands [18]. Nonetheless, host plant-specific core bacterial microbiota can also be observed across different edaphic and climatic conditions [16, 17, 19, 20] and growth stages [21].

The effect of host identity on the assembly of the rhizosphere bacterial microbiota is considered to be most pronounced in domesticated plants and different between crop host species [15, 22]. Monocotyledonous and dicotyledonous plants likely differentially shape the rhizosphere bacterial microbiota because of divergent root architecture and physiology [13, 23]. Plant host-specific acquirement of soil *Bacteria* in the rhizosphere of cereals can also be attributed to microbial functions [24, 25]. Plant host-specific acquired *Bacteria* may correlate with root secondary metabolite spectrum [25] which is often evolutionary conserved at the plant family level [26]. Hence, the comparison of crop species and their metabolic active rhizosphere bacterial microbiota is key to understand the plant host contribution to the bacterial microbiota assembly.

Previous studies compared host selection of the bacterial microbiota by crops, such as wheat, barley, maize, soybean, or oilseed rape in either glasshouse experiments or field trials. The rhizosphere bacterial microbiota of two or more crop species tend to share large fractions of microbial taxa [27]. Contrary, divergence at phylum level and overall composition have also been observed [15, 23, 24]. Moreover at a finer taxonomic resolution, *Bacteria* specific and essential to individual host crops have been recently identified [28–30]. To resolve such partially inconsistent observations regarding host-specificity of bacterial keystone taxa on the one hand and a frequent detection of many rhizosphere microbiota in different plant hosts requires an alternative and systematic assessment of their active bacterial members. These may be achieved by focusing on a core microbiota in comparative experimental studies and using the SSU RNA pool as an indicator for functionally relevant and active associations.

We suggest here to screen for host-specific *Bacteria* using linear discriminant analysis and estimating biological consistency and effect size [31]. Further permanent and conditional occupancy of *Bacteria* may have distinct ecological roles to the host [32] that we delineated using core microbiota inference [33]. Further co-occurrence network and hub node analyses are well suited to predict keystone *Bacteria* that directly and indirectly preserve microbiome structure [32].

Our study addressed the bacterial rhizosphere microbiota of four major crops grown widely in Europe and worldwide, i.e. wheat (*Triticum aestivum* L.), rye (*Secale cereale* L.), barley (*Hordeum vulgare* L.)*,* and oilseed (*Brassica napus* L.)*.* We compared the rhizosphere bacterial microbiota of single plant holobionts grown in pots and with the same agricultural soil. Two rhizosphere compartments (close, RZP; distant, RZS) were sampled to resolve niche partitioning of *Bacteria* in the rhizosphere [34]. Active *Bacteria* present among the rhizosphere microbiota were detected through the SSU RNA pool as a phylogenetic marker and proxy for activity [18, 35]. We focused on the temporal persistent *Bacteria* assuming a conserved symbiotic relationship and identified the hub *Bacteria* of individual crop host species and their direct cohort interactions partners by network comparisons to resolve differences between crop rhizosphere bacterial core microbiota irrespective of abundance.

We expected to detect rhizosphere bacterial microbiota of the three cereal crop species and oilseed rape that (a) exhibit diverging composition affected by individual plant host filtering and that (b) comprise bacterial biomarker taxa specific for each crop species within the host core microbiota. (c) We further expected that hub *Bacteria* occur among the core microbiota of each host, which foster the acquirement of unique cohort sub-networks and orchestrate differential associations as host-specific signatures.

## Methods

### Experimental design and sampling

A glasshouse experiment was conducted at Leibniz Centre for Agricultural Landscape Research (Müncheberg, Germany) with summer cultivars of wheat (Quintus), barley (Marthe), rye (Ovid), and oilseed rape (Campino) from May to July 2019. An arable soil was collected for planting during April 2019 near Prenzlau (Germany). The soil was air dried, sieved and homogenized. The soil texture was between medium loamy to medium silty sand (SI3/SU3) and the chemical characteristics are listed in Tables 1 and 2. Single plants in pots of 1.5 l were grown in a randomized design. Each pot was adjusted to a soil density of 1.2 g l^−1^ and initially watered to 60% field capacity. Seeds were sterilized with 3% bleach for 3 min and washed three times with water. The soil was fertilized in field with (100 kg ha^−1^ N, 22 kg ha^−1^ S and 27 kg ha^−1^ Mg) and received a second fertilization (N, 44 mg kg^−1^ soil) 30 days after emergence.

**Table 1:** Bulk soil chemical properties

NH_4_^−^	NO_3_^−^	pH	C_t_	N_t_	S_t_	TIC	TOC
mg g^−1^ soil	mg g^−1^ soil		%	%	%	%	%
0.0008	0.0276	5.415	0.721	0.068	0.0118	0.013	0.659

**Table 2:** Bulk soil chemical properties of plant available cations

*P*	*K*	*Na*	*Mg*	*Ca*	KAK_pot_
mg g^−1^ soil	mg g^−1^ soil	mg g^−1^ soil	mg g^−1^ soil	mg g^−1^ soil	cmol g^−1^
0.0608	0.1150	0.0042	0.0166	0.5479	5.818

Four replicate samples of the initial planting soil were included in the analysis (Additional file 1: Table S1). During booting, flowering and milk ripening five replicate plants were sampled per crop species. Bulk soil was removed by slicing up the pot and tapping on the table. Thus, the soil aggregates were broken and loosely attached soil fell off when carefully shaking the root system. Two separate rhizosphere fractions were obtained: Distant rhizosphere (RZS) was collected by vigorous shaking and holding the plant at the stem, without hurting the root tissue. Close rhizosphere (RZP) refers to the soil layer remaining after mechanical collection of RZS. This fraction was recovered by placing the root system into 50 ml tube with PBS buffer (pH 7.4), and putting on a rotary shaker (125 rpm, 5 min). The detached soil suspension was than centrifuged at 4500×*g* for 10 min to sediment the rhizosphere. All samples for molecular analysis were frozen in liquid nitrogen and stored at −80 °C until further processing.

### Soil chemical parameters

The analysis of soil chemical parameters (Tables 1, 2) was performed at the facility ‘Zentrallabor ZALF Müncheberg’ following the methodology of respective DIN standard for ammonium, nitrate, soil pH, total carbon (Ct), total nitrogen (Nt), total sulfur (St), total inorganic carbon TIC and total organic carbon (TOC) or standard protocols recommended by the Association of German Agricultural Analytic and Research Institutes (VDLUFA) e.V.

### RNA extraction and purification

RNA was extracted using a protocol adapted from two previously described protocols (Griffiths et al., 2000; Töwe et al., 2011). Briefly, 0.4 g rhizosphere sample were mixed in a bead tube (PowerBead Pro Tubes QIAGEN®) with equal amounts of Hexadecyltrimethylammonium bromide (CTAB; 10% (wt/vol)) extraction buffer (240 mM potassium phosphate, 0.7 M NaCl, 10 μl ml^−1^ ß-mercaptoethanol, pH 8) and phenol-isoamyl-chlorophorm with volumetric ratio of 25:24:1). Homogenization was performed twice for 40 s at a speed of 5.5 m s^−1^ in a bead mill (MP Biomedicals™ FastPrep-24 Instrument) interrupted by inverting the tubes for two minutes to avoid heat degradation. Followed by centrifugation for (20 min, 4 °C 16,100×*g*). Resulting aqueous raw extract was recovered and purified twice with chloroform:isoamylalcohol (with volumetric ratio of 24:1). Subsequently, RNA was precipitated with 10% polyethyleneglycol 6000 while being incubated for 90 min on ice. The precipitate was recovered by centrifugation (30 min, 4 °C, 16,100×*g*) and purified by washing with ice cold 70% ethanol. Subsequent to centrifugation (5 min, 4 °C, 16,100×*g*) the RNA pellet was suspended in 80 µl RNAse-free water.

### RNA purification and cDNA synthesis

The extract was digested with DNAse (TURBO DNA-free™ Kit, Invitrogen™) to recover pure RNA extract. Obtained RNA concentration was measured at a fluorometer with a selective RNA binding dye (Qubit™ RNA BR Assay Kit). RNA quality was ensured with a value of two or higher in optical density ratio OD 260/280. Total RNA (200 ng) were than transcribed into cDNA (Biozym cDNA Synthesis Kit) within the same day. Pure RNA extracts were checked for residual bacterial DNA by 16S rRNA PCR amplification (see ‘PCR for amplicon sequencing preparation’). Absence of DNA, i.e. lack of an amplicon, was proven by subsequent gel electrophoresis and only such checked RNA solutions were used for further steps.

### PCR for amplicon sequencing preparation

Bacterial composition was analyzed by amplicon sequencing of the reverse transcribed 16S rRNA as phylogenetic marker. The hypervariable V4 region was targeted using the primers 515F-806R [36, 37] with terminal universal adapter sequences to link DNA barcodes and sequencing adaptors. 50 µl PCR consisted of GoTaq® G2 Hot Start Master Mix, primer (0.1 µM) and 4 µl cDNA template (1:50 dilution). The thermal program was 95 °C for 120 s, 25 cycles of 95 °C for 40 s, 56 °C for 30 s, 72 °C for 40 s and a final elongation at 72 °C for 300 s. Amplicon size homogeneity was verified by gel electrophoresis. A pooled negative control of all PCR runs was included. Further library preparation and 300 bp paired-end read Illumina Miseq V3 sequencing were performed by LGC genomics GmbH Berlin.

### Bioinformatic pipeline for analysis of amplicon sequencing dataset

Demultiplexing was conducted with Illumina bcl2fastq 2.17.1.14 software following clipping of barcode and sequencing adapters. Primer were removed using Cutadapt v3.0 (Martin, 2011) following sequence processing using QIIME 2 v2020.8 [38]. Denoising was performed by applying the build in method for DADA2 [39] with forward and reversed reads truncated at 250 bp and 200 bp, respectively. Amplicon sequencing variants (ASV) were assigned to taxonomy using the naïve bayesian classifier for Silva 138. 99%-OTUs from 515F/806R region of sequences [40, 41]. A phylogenetic tree was generated using IQ-TREE 2 [42]. The pipeline started from 14,819,213 singles reads and yielded 10,810,699 non chimeric sequences, which corresponds to an average recovery of 85,799 sequences or 70% per sample (n = 125). Subsequent to removal of unidentified taxa beyond species level and plastid sequences, 11,710 non-singleton AVSs were obtained, of which 6817 occurred in more than one sample. These belonged to 36 bacterial phyla and 590 genera.

### Statistical and network analysis

Statistical analysis was conducted in R v3.6.0-4.0.0 [43] and Rstudio [44]. Filtering and storage of sequencing data were carried out using phyloseq [45] and visualization were produced by ggplot2 [46] and circilize [47]. Alpha diversity was calculated using the inverse Simpson index, observed ASVs and Shannon index based on rarefactioned counts to 10^4^ reads per sample (Additional file 1: Figure S1). Multivariate statistics namely Principal coordinates analysis (*PCoA*) and Permutational analysis of variance (*PERMAOVA*) to test the effect of crop species, plant growth stage, and rhizosphere compartment on overall variance of the bacterial microbiota composition were conducted using the ordinate and adonis2 function of the vegan package [48]. *PCoA* analysis was computed based on weighted UniFrac distance. Core bacterial microbiota of the close and distant rhizosphere for each of the four crop species were computed and defined as ASVs occurring at all three growth stages and in all four replicates (n = 3 × 5).

Linear discriminant analysis effect size (*LEfSe*) [31] was applied to identify biomarker taxa explaining differences between core bacterial microbiota of the crop species and both rhizosphere compartments (RZP, RZS). Co-occurrence networks of the eight core bacterial microbiota were computed with the SPRING model [49]. Network analysis was performed with the NetCoMi package [50]. Hubs were identified as nodes above the 95% quantile of the fitted log-normal distribution of the three normalized network metrics degree, betweenness and closeness centrality. Hubs were further investigated regarding their direct associations referred to as cohort nodes. Network comparison required to subset to ASVs present in both core bacterial microbiota under investigation. Differential associations were verified using the discordant method [51].

## Results

### Niche partioning and partially host and growth stage explain rhizosphere bacterial microbiota variation

The variation in the bacterial microbiota considering all plant hosts and growth stages was mainly affected by spatial differentiation in the rhizosphere as indicated by 21% of variance explained (*R*^2^) by contrasting close and distant rhizosphere (PERMANOVA). Additionally, both rhizosphere compartments were significantly different from the initial bulk soil (Table 3). Crop species and growth stage accounted for 10% and 6% respectively, with all factors being statistically significant (*P* < 0.001) (Table 4).

**Table 3:** Divergence of rhizosphere from initial planting soil bacterial microbiota composition

Factor	Df	SumOfSqs	*R*^*2*^	*F*	P*
Rhizosphere vs bulk soil	2	0.645	0.304	23.611	** < .001**
Residual	108	1.476	0.706		
Close rhizosphere vs. bulk soil	55	0.172	10.204	114.11	** < .001**
Residual	56	0.669	0.796		
Distant rhizosphere vs. bulk soil	56	0.268	0.245	18.157	** < .001**
Residual	57	0.826	0.755		

Permutational analysis of variance and weighted UniFrac distance

^*^Bold font: factors considered significant

**Table 4:** Influence of rhizosphere compartment, crop species, and growth stage on entire and core bacterial microbiota

Factor	Df	SumOfSqs	*R*^*2*^	*F*	P*
**Full dataset (close & distinct rhizosphere)**					
Growth stage	2	0.108	0.057	4.969	** < .001**
Crop species	3	0.188	0.100	5.838	** < .001**
Rhizosphere (close&distant)	1	0.425	0.225	39.53	** < .001**
Growth stage:crop species	6	0.156	0.083	2.421	** < .001**
Residual	94	1.01	0.535		
**Core bacterial microbiota distant rhizosphere**					
Growth stage	2	0.081	0.020	8.398	** < .001**
Crop species	3	3.694	0.918	253.680	** < .001**
Growth stage:crop species	6	0.031	0.008	1.076	0.394
Residual	0.218	0.054			
**Core bacterial microbiota close rhizosphere**					
Growth stage	2	0.119	0.030	8.842	** < .001**
Crop species	3	3.384	0.851	166.968	** < .001**
Growth stage:crop species	6	0.195	0.049	4.807	** < .001**
Residual	41	0.277	0.070		
**Bacterial microbiota distant rhizosphere**					
Growth stage	3	0.380	0.347	14.685	** < .001**
Crop species	3	0.199	0.182	7.698	** < .001**
Growth stage:crop species	6	0.127	0.116	2.454	** < .002**
Residual	45	0.388	0.355		
**Bacterial microbiota close rhizosphere**					
Growth stage	2	0.075	0.115	4.209	** < .001**
Crop species	3	0.084	0.129	3.150	** < .001**
Growth stage:crop species	6	0.126	0.195	2.370	** < .001**
Residual	41	0.365			

Based on permutational analysis of variance and weighted UniFrac distance

Core bacterial microbiota of wheat, barley, rye and oilseed rape includes ASVs present within each replicate per crop species and at every growth stage (booting, flowering, ripening)

^*^Bold font: factors considered significant

**Fig. 1:**
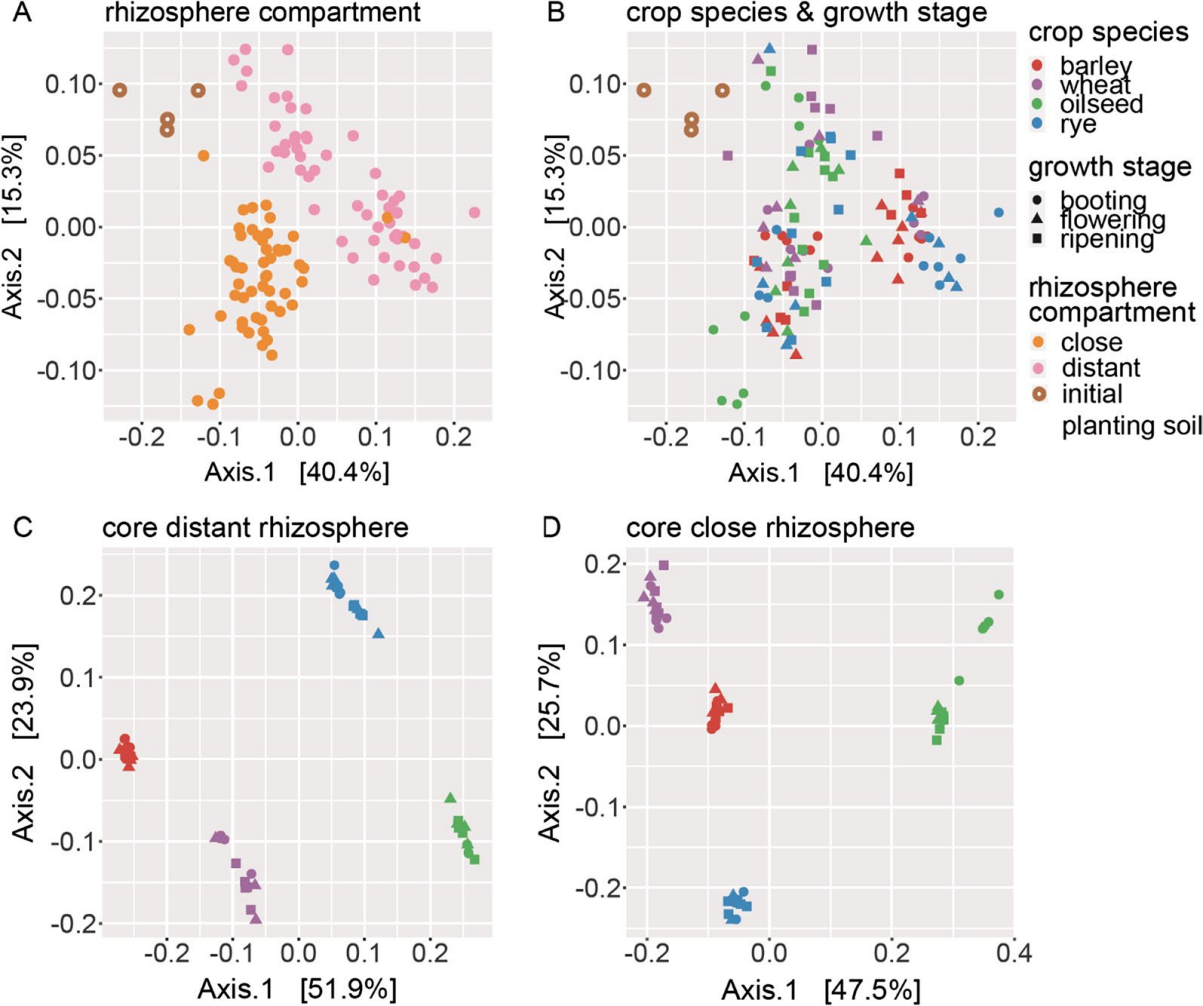
Principal Coordinates Analysis using weighted UniFrac distance for unfiltered bacterial microbiota **A** by rhizosphere compartment, **B** by crops species and growths stages and for the core rhizosphere microbiota of wheat, barley, rye and oilseed rape for the **C** distant and **D** close rhizosphere

Looking at the individual compartment, we observed a stronger growth stage effect in the distant (*R*^*2*^ = 34%) than in close rhizosphere (*R*^*2*^ = 11%). Crop species captured a significant proportion of microbiome variation, which was around 18% and 12% in the distant and close rhizosphere, respectively. We found a significant interaction between plant growth stage and crop species, which explained an additional 12% of variation in both rhizosphere compartments. This suggests a differential response of the bacterial microbiota associated with a specific plant growth stage in different crop host species. PCoA on weighted UniFrac distance resembled the separation of the close from the distant rhizosphere as primary effect (Fig. 1A, B). Bacterial microbiota assembly of cereal species and oilseed rape diverged at booting, but tended to converge at mid and late growth stages. Contrastingly, barley and oilseed rape were temporally invariant within the distant rhizosphere and formed separate clusters. Rye and wheat associated with barley at booting and diverged between flowering and late growth stages. Hence, the distance to oilseed rape decreased implying a temporal shift in microbiome structure.

### The core rhizosphere bacterial microbiota encompassed growth stage-invariant *Bacteria* enriched within individual crop species

Host-species effect signatures within the rhizosphere bacterial microbiota were inferred from eight core bacterial microbiota for each host species separated into close and distant rhizosphere (Additional file 1). The core bacterial microbiota gathered from joint datasets of all four crop species represented more than half of summed ASV counts (RZP 58%, RZS 50%), but were limited to a low fraction of ASVs (8%) (Table 5).

**Table 5:** Number and relative abundance of ASVs included in the host crop core microbiota

Rhizosphere compartment	Wheat	Barley	Rye	Oilseed rape	Agglomerated
**Distant**					
ASVs	283	345	154	122	453
ASVs (% of total)*	4.6	5.6	2.5	2	8.1
ASV count (% of total)*	11.6	12.8	8.3	8.4	50.2
**Close**					
ASVs	446	296	203	137	512
ASVs (% of total)	7.0	4.8	3.3	2.2	8.3
Sequence count (% of total)	21.0	13.0	9.2	15.0	58.0

^*^Proportion of ASVs and ASV counts in relation to the unfiltered microbiota

This implies, that a low number of core *Bacteria* were the dominant microbiota associated with the four crops studied. Moreover, PCoA analysis demonstrated an uniform clustering by crop species within the core bacterial microbiota independent of growth stage (Fig. 1C, D), with the host plant effect explaining almost the entire variation (PERMANOVA, R^2^ = 92 RZS, R^2^ = 82 RZP). Thus, the core bacterial microbiota derived in our study encompassed plant host-specific *Bacteria*. These originate from a typical arable soil and can be considered as non-transient host traits, since they persisted over growth stages.

**Fig. 2:**
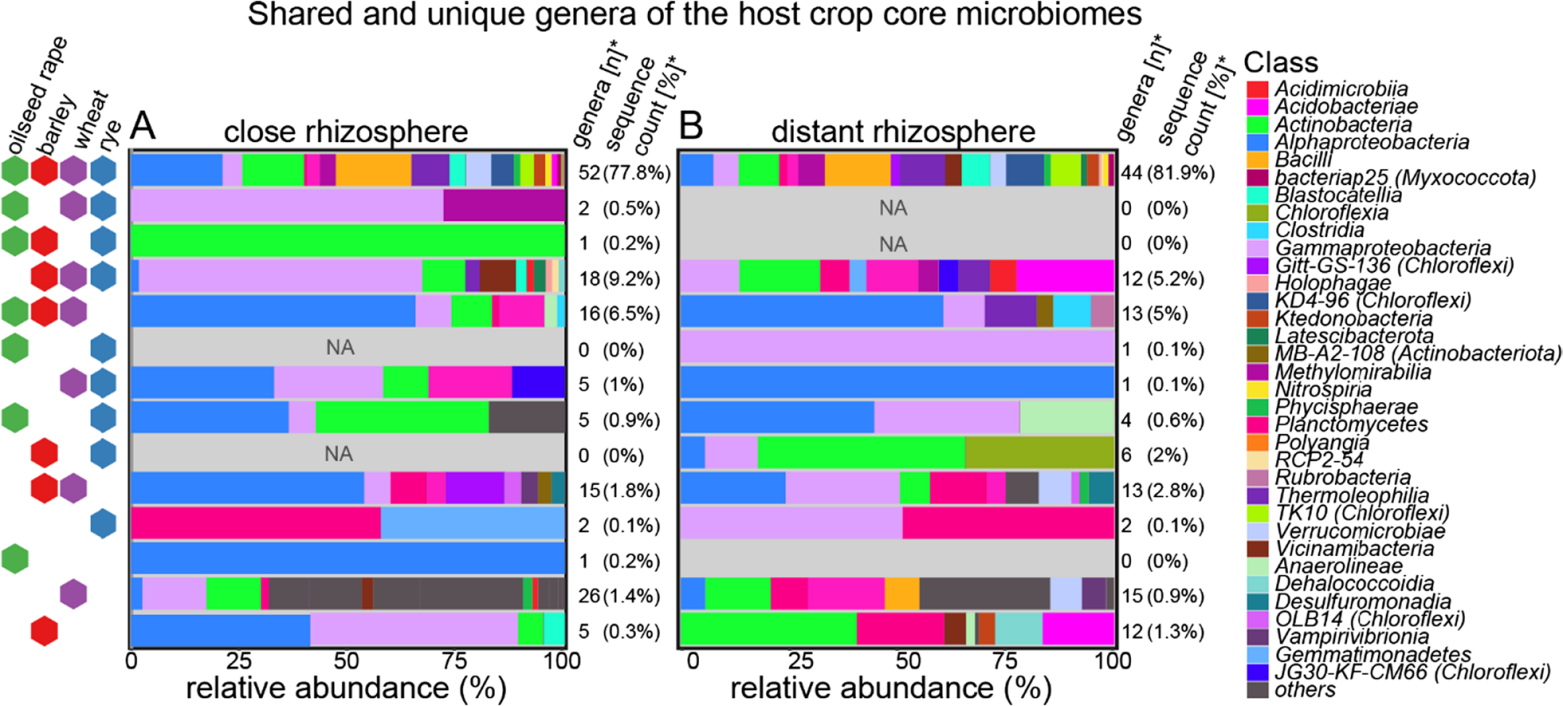
Shared and unique bacterial genera present in the core rhizosphere microbiota of wheat, barley, rye and oilseed rape of the **A** close and **B** distant rhizosphere. Each row corresponds to a venn diagram fraction described by the left hand key, *n: total number of genera. **sequence count (%): sequence percentage of total

For taxonomic description, the core bacterial microbiota were aggregated at the genus level. Generally, more than two genera unique to each crop species (ratio RZS:RZP) occurred in wheat (15:26) and barley (12:5), i.e. with a total abundance below 1.5% of total sequences count within the core microbiota (Fig. 2). Thus, unique genera were scarce and might belong to the rare species pool. Contrastingly, the 52 (distant rhizosphere) and 44 (close rhizosphere) genera that occurred in all four crop core bacterial microbiota of the respective rhizosphere compartment were highly abundant (about 80% of total sequences count), meaning that this comparable small subset were dominant traits of the core microbiota. Additionally, more than ten genera were shared between the three cereals or between barley, wheat an oilseed rape thereby contributing to more than 5% of the total sequences counts within the aggregated core microbiota. As a consequence, enriched or depleted genera were the most abundant core microbiota members for each of the four crop species, while unique bacterial genera were scarce.

The aggregated core microbiota were predominantly composed of the genera of the classes *Alpha-* and *Gammaproteobacteria, Actinobacteria*, *Thermoleophilia*, *Bacilli*, *Verrucomicrobia*, unclassified *Chloroflexi* of the group *‘*KD4*-*96’*,* which together accounted for more than 50% of relative abundance. *Proteobacteria* were more abundant within the close rhizosphere, whereas genera of the *Thermoleophilia* and ‘TK10’ were lower abundant than in distant rhizosphere*.* Genera of the class *Bacilli* were substantially enriched (25%) in oilseed rape close rhizosphere. Exclusive bacterial genera of barley belonged mainly to the Alpha- and *Gammaproteobacteria* (close rhizosphere) or *Actinobacteria, Acidobacteria, Planctomycetes* (distant rhizosphere). In contrast to barley, genera of the *Bacilli* and *Polyangia* were uniquely present in the distant rhizosphere of wheat.

Genera exclusively shared between barley, wheat, and oilseed rape belonged mainly to the *Alpha-* and *Gammaproteobacteria* and were characterized by the presence of *Rubrobacteria* and *Clostridia*. Remarkably, genera exclusively found in cereals were mainly *Gammaproteobacteria* but no *Alphaproteobacteria*.

The primary result of both core inference and venn diagram partitions was that shared genera between all crop species and triplicate comparison incorporate most of the of the total sequences counts of the aggregated core microbiota. In contrast, unique genera of the crop species microbiota exhibited low aggregated sequence counts.

### *LEfSe* biomarker analysis

**Fig. 3:**
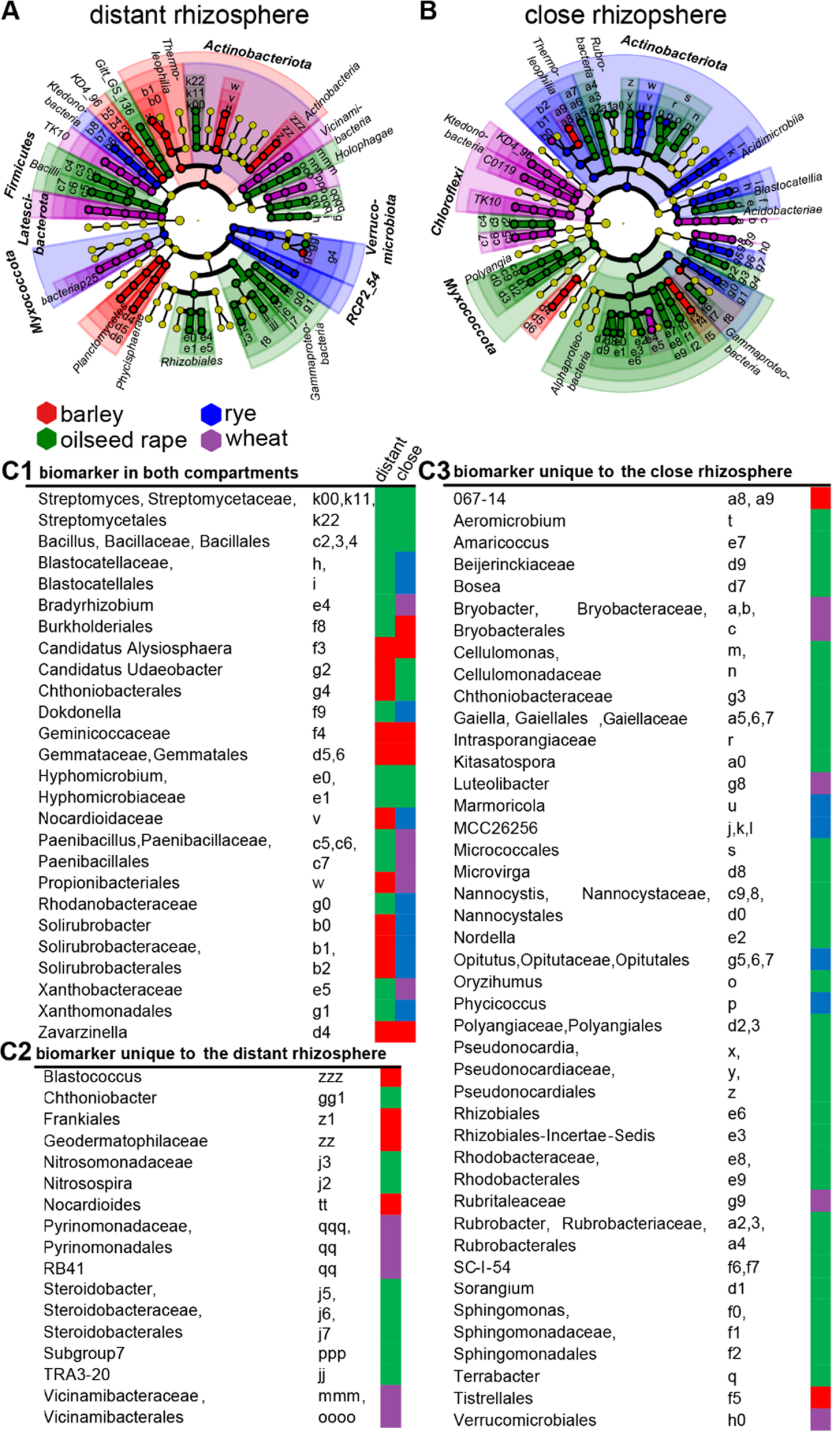
Biomarker taxa of wheat, barley, rye and oilseed rape bacterial core microbiota at the genus rank derived from the close and distant rhizosphere identified by linear discriminant analysis effect size (LEfSe**) visualized as circular taxonomic trees. **A** distant rhizosphere; **B** close rhizosphere; **C1** Biomarker from order to genus rank occurring in both rhizosphere compartments; **C2** Biomarker from order to genus exclusive to the close rhizosphere; **C3** Biomarker from order to genus exclusive to the distant rhizosphere **LEfSe: Linear discriminant analysis effect size

Most genera were present across core bacterial microbiota of all crop species. Hence, we identified biomarker taxa from genus to phylum rank among them using *LEfSe* analysis. These biomarker were the dominant bacterial phylotypes associated with a specific crop species and were the main taxa that explained differences between the core bacterial microbiota of the four crops species (Fig. 3 and Table 6).

**Table 6:** Global co-occurrence network metrics for each core microbiota of the crop species and rhizosphere compartment

Niche	Crop species	Nodes	Clustering coefficient	Modularity	Positive edges (%)	Average degree	Average betweenness	Natural connectivity
Close	Barley	107	0.083	0.563	50	0.035	0.028	0.011
	Wheat	139	0.098	0.483	56	0.036	0.017	0.009
	Rye	77	0.119	0.608	52	0.036	0.048	0.015
	Oilseed rape	71	0.156	0.604	51	0.041	0.043	0.016
Distant	Barley	99	0.091	0.512	52	0.040	0.027	0.012
	Wheat	102	0.087	0.530	55	0.039	0.028	0.012
	Rye	61	0.133	0.645	51	0.040	0.054	0.018
	Oilseed rape	58	0.089	0.677	47	0.038	0.078	0.019

Additionally, we determined bacterial genera characteristic of spatial differentiation between the close and distant rhizosphere based on the entire dataset irrespective of the crop species (Additional file 1: Figure S2).

One third of the biomarker taxa occurred in both rhizosphere compartments but were indicative of two different crop species (Fig. 3C1). Among them several lower taxonomic groups of the *Gammaproteobacteria* were indicative of oilseed rape within the distant rhizosphere, whereas these were a biomarker taxon of rye or barley in the close rhizosphere. The families *Blastocatellia* and *Dokdonella* were biomarker taxa of rye in the close rhizosphere and belonged to oilseed rape in the distant rhizosphere. Accordingly, several biomarker that were assigned to oilseed rape in the distant rhizosphere, were assigned to wheat within the close rhizosphere, e.g. *Paenbacillus* or *Bradyrhizobium* Additionally, several biomarker taxa of a crop species bacterial microbiome identified in the close rhizosphere became non-discriminative in the distant rhizosphere. This was most apparent for biomarker taxa belonging to the order *Rhizobiales*. Interestingly, *Bacillus* and *Streptomyces* were biomarker of oilseed rape in both compartments. Further, the bacterial microbiota of barley tended to show identical biomarkers in both rhizosphere compartments, such as the family *Gemmataceae* or the genus *Candidatus Alysiosphaera.*

Biomarker of rye belonged uniformly to the close rhizosphere, e.g. *Optitutaceae.* The majority of biomarker taxa exclusively derived from the close rhizosphere belonged to the bacterial microbiota of oilseed rape such as *Sphingomonas* and *Pseudnarcodia* (Fig. 3B, C3). *Verrucomicrobiales* were a unique biomarker of wheat within the close rhizosphere. Biomarker exclusively detected in the distant rhizosphere that belonged to barley were among other the order *Frankiales* and *Nocardioides.*

Thus, the rhizosphere compartment essentially determined biomarker taxa assignment. Thereby, we observed assignments of the same biomarker taxa to different crop species only differing by the two rhizosphere compartments. This required further investigations of the identified individual biomarker genera and the underlying differences between hosts core microbiota, they were characteristic of. Thus, we examined co-occurrence patterns of the core bacterial microbiota to confirm host specificity and the structural importance of so far identified biomarker genera.

### Network hubs of the crop core bacterial microbiota

We performed co-occurrence network analyses for each core bacterial microbiota visualized as chord diagrams to identify structurally important interactions within the individual crop bacterial microbiota in separated data sets of the close and the distant rhizosphere (Additional file 1: Figures S3, S4).

The size of the largest connected component was 1.5-fold larger in wheat and barley, compared to rye and oilseed rape (Table 5). Higher average degree, betweenness and natural connectivity were observed in all networks of the close compared to the distant rhizosphere. Similarly higher average degree, betweenness and natural connectivity were observed for the networks of rye and oilseed rape compared to barley and wheat (Table 5). Accordingly, the network structure of wheat and barley was more dependent on individual nodes compared to the other host plant species.

**Fig. 4:**
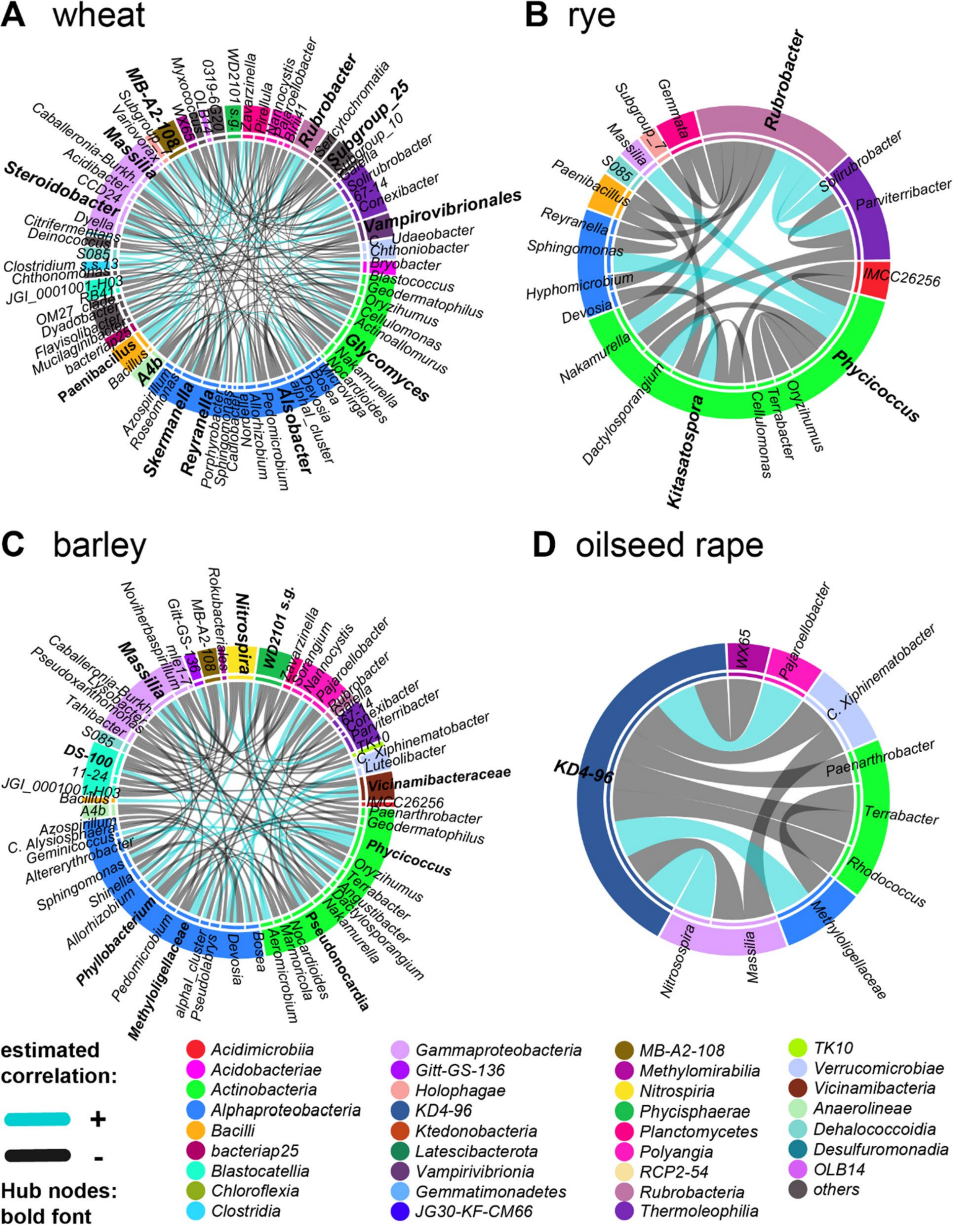
Co-association networks of the close rhizosphere core bacterial microbiota of wheat, rye, barley and oilseed rape visualized as chord diagram reduced to hub nodes (bold font) and their cohort partners

**Fig. 5:**
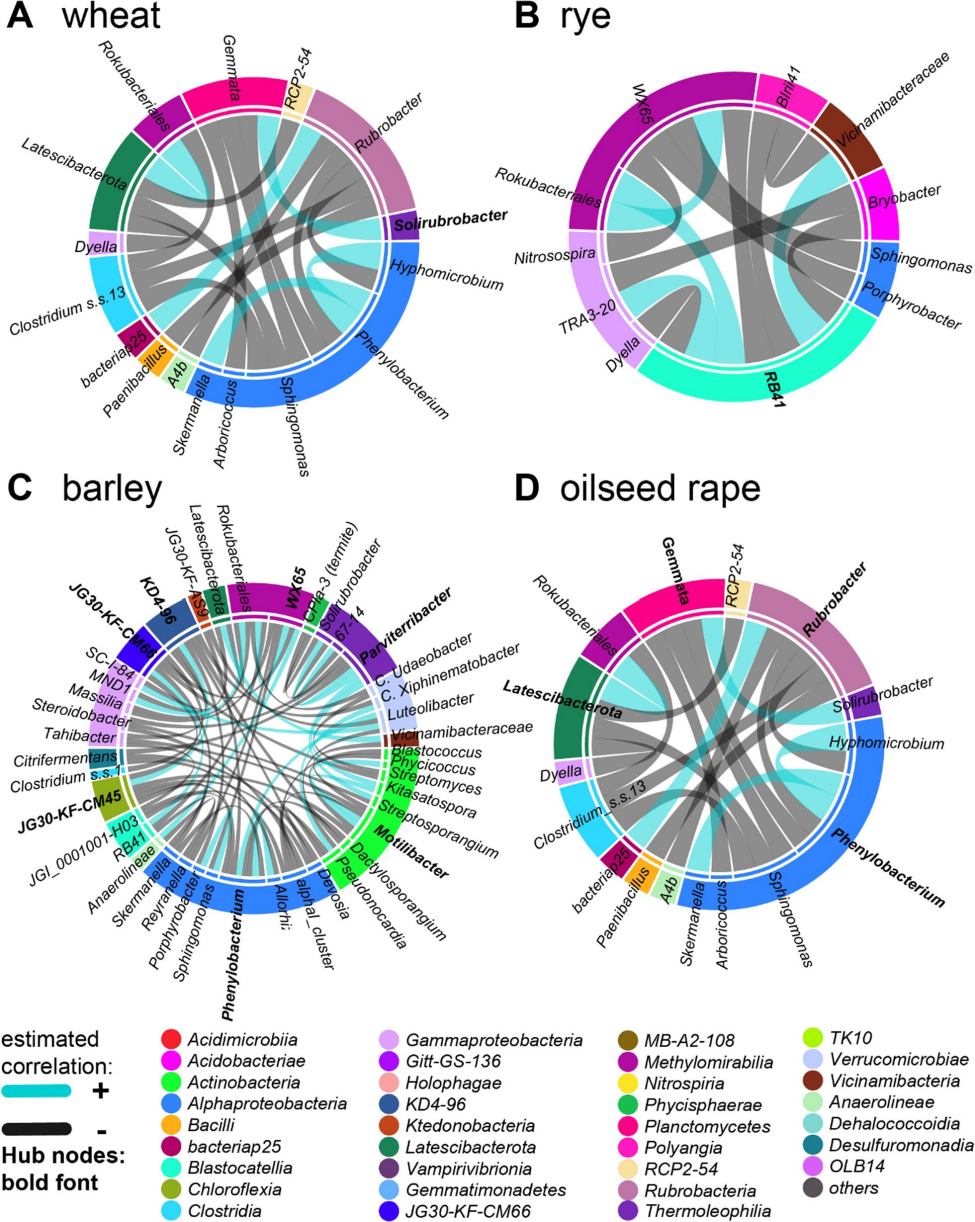
Co-association networks of the distant rhizosphere core bacterial microbiota of wheat, rye, barley and oilseed rape visualized as chord diagram reduced to hub nodes (bold font) and their cohort partners

More than ten hubs were identified for wheat and barley within the close rhizosphere, which interconnected almost the entire network (Fig. 4, 5), while rye and oilseed rape comprised less than four hubs that were directly associated with less than 16 cohort nodes (Figs. 4, 5). The networks of wheat, rye and oilseed rape within the distant rhizosphere comprised less than three hubs, which directly connected only a small subset of cohort nodes. In contrast, the seven hubs of barley form a complex subnetwork with their cohort nodes similarly to the close rhizosphere. As a result, the co-occurrence networks of (a) rye and oilseed rape as well as of (b) wheat and barley are structurally more similar to each other, respectively.

Hubs shared between at least two cereal crop species in the close rhizosphere were *Massilia, Rubrobacter,* and *Phycicococcus.* Thereby*,* more than three hubs of barley and wheat belonged to the class *Alphaproteobacteria*. Hubs of wheat, barley and to a lower extent of rye associated with similar nodes. Individual hubs of oilseed rape were unclassified *Chloroflexi* group *‘*KD4*-*96’, *Phenylobacterium*, and *Rubrobacter*. Exclusive hubs of wheat were among others *Steroidobacter, Glycomyces, Vampirovibrionales*, *Clostridium ss.13* and *Solirubrobacter*. The only hubs exclusively found in rye were *Kitasatospora* and unclassified *Pyrinomonadaceae* ‘RB41’*.* Remarkably, *Nitrospira* was an exclusive hub of barley, which was not associated with cohort nodes in the other crop species (Fig. 4).

### Differentially associated nodes of the bacterial rhizosphere core microbiota

**Fig. 6:**
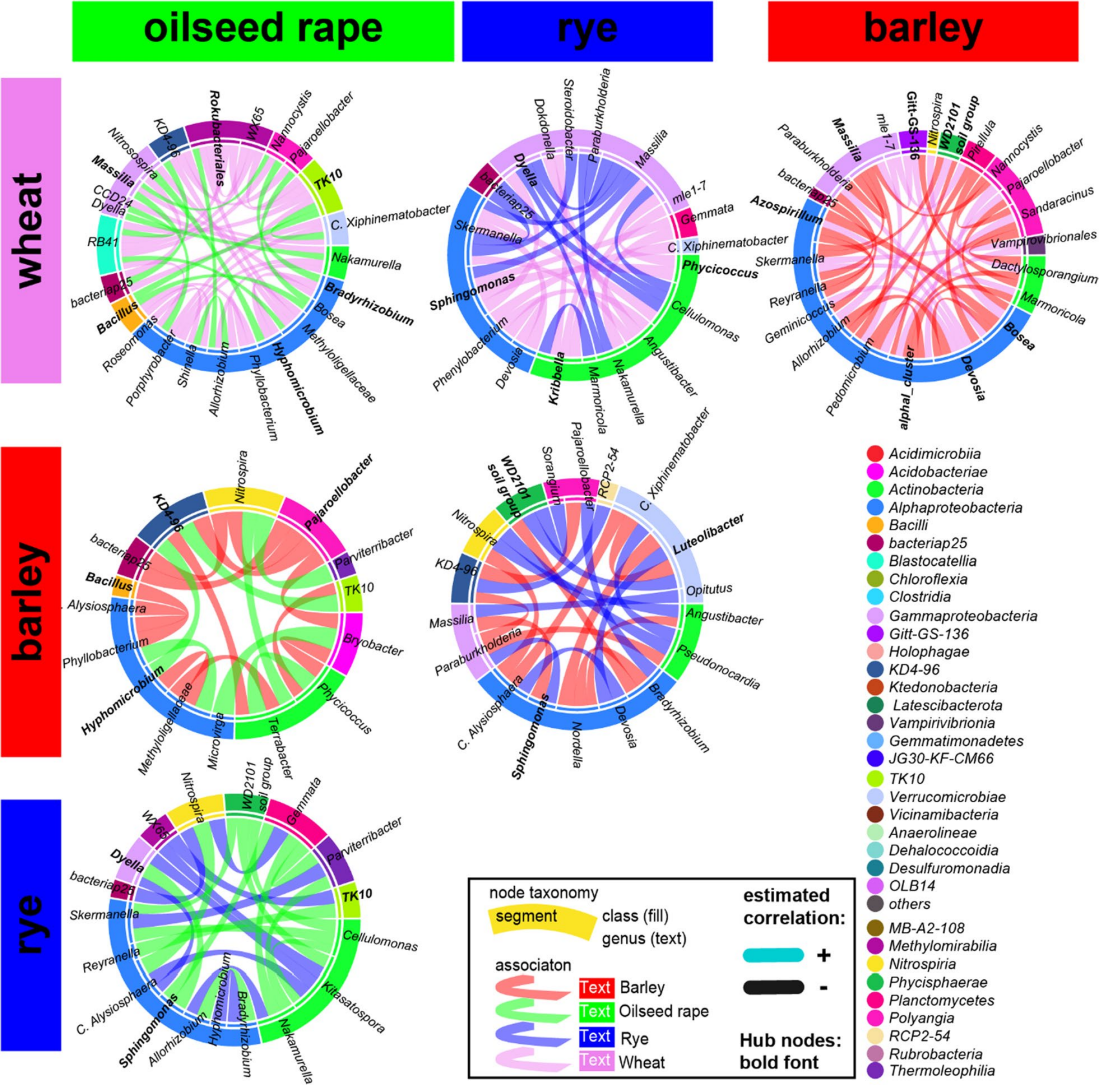
Matrix of pairwise comparison of wheat, barley, rye and oilseed rape co-association networks of the close rhizosphere visualized as chord diagrams with only significant different association included and colored by the respective crop bacterial microbiota in which it occurs

**Fig. 7:**
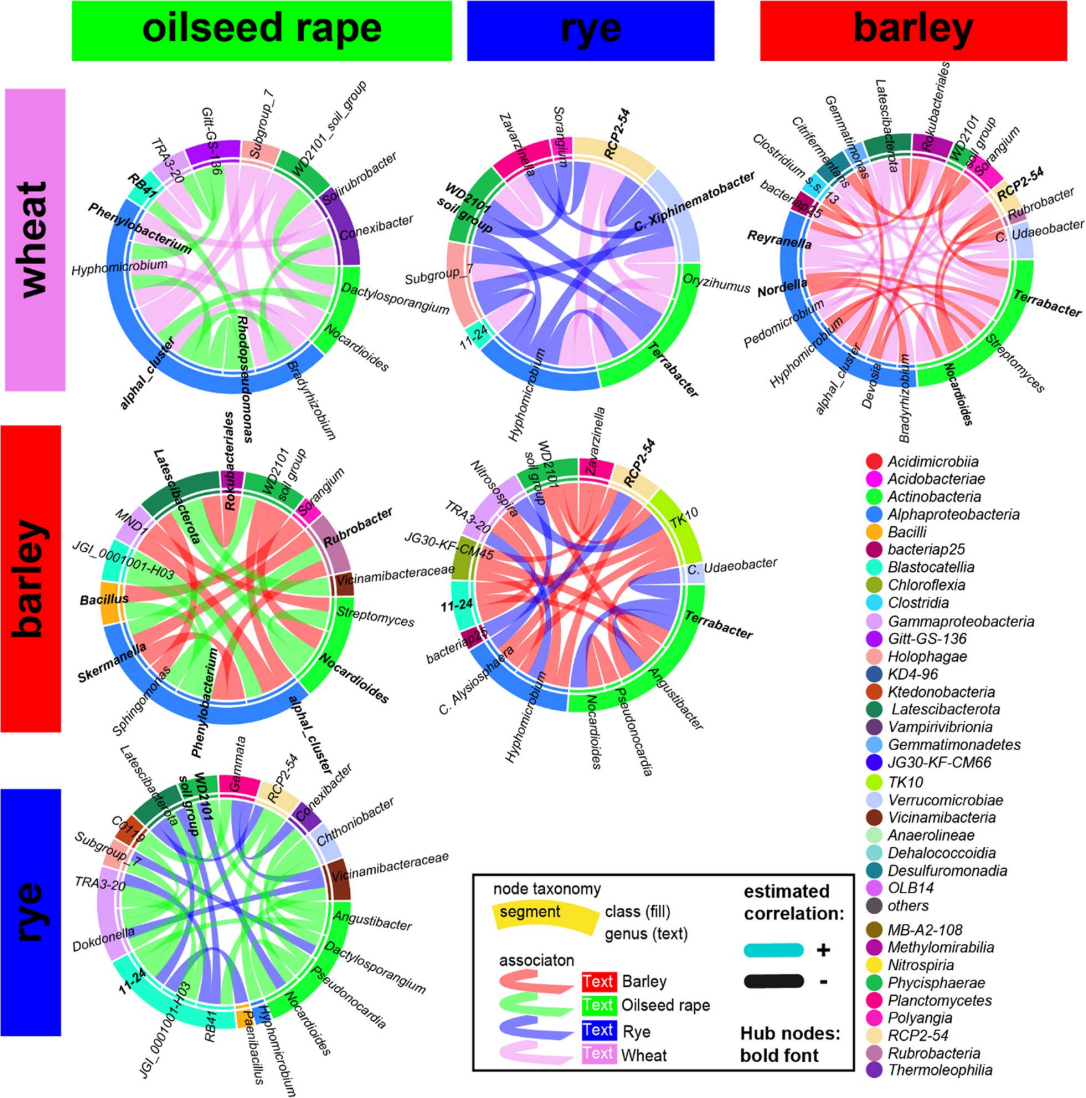
Matrix of pairwise comparison of wheat, barley, rye and oilseed rape co-association networks of the distant rhizosphere visualized as chord diagrams with only significant different association included and colored by the respective crop bacterial microbiota in that it occurs

We examined pairwise significant differences in associations of shared genera between each crop core bacterial microbiota to demonstrate that the core taxa across crop species distinctively influence the bacterial microbiota structure and assembly. In most comparisons (Figs. 6, 7), half of the differentially associated nodes belonged to the *Alphaproteobacteria* and *Actinobacteria.* The most frequent and differentially associated genera (Table 7) were unclassified *Tepidisphaeraceae* ‘WD2101 soil group’*, Hyphomicrobium, Terrabacter,* uncultured *Beijerinckiaceae* ‘alphaI cluster’*, Nocardioides, Massilia,* and *Bradyrhizobium.*

**Table 7:** Most frequent* significant differential associated nodes within pairwise comparisons of the crop species** core bacterial microbiota co-association networks

Rhizosphere	Number of differential associations	
	Close	Distant
*WD2101_soil_group*	32	26
*Hyphomicrobium*	31	23
*Terrabacter*	28	21
*alphaI_cluster*	27	19
*Skermanella*	NA	17
*Nocardioides*	25	NA
*Nitrospira*	21	NA
*Massilia*	21	17
*TK10*	NA	15
*Bradyrhizobium*	21	14
*Devosia*	NA	14

^*^0.9 percentile

^**^Barley, wheat, rye and oilseed rape

While *Bradyrhizobium* was identified as a hub of oilseed rape, the association of *Bradyrhizobium* and *Hyphomicrobium* was specific to the three cereals. Another specific feature of cereals was the association of *Nocardioides* and uncultured *Beijerinckiaceae* ‘alphaI cluster’ (only wheat and barley) with unclassified *Tepidisphaeraceae* of the group ‘WD2101 soil group’*,* while in oilseed rape *Nocardioides* formed associations with other genera of the *Actinobacteria,* such as *Streptomyces.* Differential associations contrasting the individual cereal crop species were formed by *Cellulomonas* and *Skermanella* as well as unclassified *Pyrinomonadaceae* ‘RB41’ for rye. Significant different association specific to wheat and barley were guided by *Massilia, Sphingomonas, Bosea, Devosia* and *Candidatus Xiphinematobacter* as well as *Optitutus* in rye. Thus, a limited set of genera promoted different microbiome structures between the crop species. These shared genera were indicative of differences between cereal crops and oilseed rape and between individual crop species. Moreover, cereal hosts tended to associate with similar nodes compared to oilseed rape.

As a result, co-occurrence network analyses explained differences in rhizosphere microbiome assembly between plant families as well as individual crop species. Thus, cohort subnetworks and differential associations elucidated a host species effect that was not evident from differential abundance of specific bacterial genera enriched or unique to a specific plant host.

## Discussion

A glasshouse experiment was conducted to investigate if host species-specific keystone *Bacteria* persist within the metabolically active rhizosphere bacterial microbiota of typical crop plant hosts. We demonstrated a general plant rhizosphere effect. Subsequently, crop-specific core microbiota were derived to delineate their keystone *Bacteria* based on co-occurence networks. Differential associations of the core microbiota of each crop species were resolved that comprehensively characterize host signatures within the rhizosphere microbiota.

### Plant host filtering had a subsidiary effect

A robust rhizosphere effect [15] was observed for all four crop species resulting in rhizosphere bacterial microbiota distinct from the initial soil microbiota composition and profound contrasts between the close and distant rhizosphere compartment were confirmed. This implies that root traits and activity had a different spatial impact on the bacterial microbiota structure, irrespective of plant species. However, the distant rhizosphere bacterial microbiota of oilseed rape remained more similar to the bacterial microbiota of the initial bulk soil. In contrast, the bacterial microbiota of oilseed rape within the close rhizosphere exhibited the most pronounced crop species effect. This suggests that the rhizosphere effect reached shorter than compared to the monocot cereal species. *Poaceaes* and *Brassicaceae* differ in root anatomy and growth regulation [52, 53]*.* Moreover, the production of glucosinolates typical to *Brassica* [54, 55] might have inhibited some microbiota that were not affected by cereal crop species’ roots. A mechanistic analysis is not the scope of the current study. However, the rhizosphere compartment ultimately determined rhizosphere microbiota composition, which is essential to future RNA- and transcriptome-based study designs.

Crop species and developmental impact on the bacterial microbiota were tightly coupled within the close rhizosphere as implied by a significant effect in interaction of crop species and growth stage in *PERMANOVA* analysis. The explained variance was larger than previously reported when comparing plant genotypes [15], but the crop species effect alone had only a low impact compared with previous field studies that involved cereal crop species [30]. Our study accessed only the metabolic active part of the bacterial microbiota that may per se respond more pronouncedly to host temporal variability in root metabolism. Rhizosphere-competent microbiota are generally characterized by both fast growth and rapid upregulation of gene expression [56]. Thus, they can better adapt to conserved metabolic dynamics of the plant during growth [57]. Consequently, the rhizosphere bacterial microbiota assembly was clearly linked to plant host species traits. In agreement with previous studies, the plant host effect was spatiotemporally dynamic and subsidiary to the general rhizosphere effect.

### Host species-specific genera occur in the core rhizosphere bacterial microbiota

Accounting for the assembly effect of soil and host factors in the rhizosphere [58], we derived core bacterial microbiota of oilseed rape, barley, rye and wheat, respectively. In turn, microbiome assembly patterns were affected only by host plant species-specific acquirement, since the variation of the merged core bacterial microbiota was almost entirely explained by the factor crop species*.* Prevalent bacterial taxa belonged to the shared fractions. This suggests a refinement from an established rhizosphere instead of parallel acquirement from bulk soil, which would have led to host plant-unique identities of bacterial taxa. Hence, the two-step selection model [10] applies to our results that postulates a host fine-tuning of the rhizosphere bacterial microbiota.

Generally, in our study we observed an increased importance of *Gammaproteobacteria* in the bacterial microbiota of cereals. The taxonomic composition of the core bacterial microbiota broadly covers *Bacteria* found in previous studies. These examined the plant host effect on rhizosphere microbiome of wheat, oilseed rape, and further cereals with reduced environmental settings [16, 21, 52, 59, 60], or as field studies [27, 34].

We identified *LEfSe* biomarkers explaining differences between the four core bacterial microbiota of crop species. *Paenibacillus*, *Verrucomicrobiales,* and *Bradyrhizobium* [59] or *Rhodanobacteriacea* [27] were associated with wheat and oilseed rape in contrast to findings of previous studies. [27, 59]. Further, *Bacillus* was characteristic to both rhizosphere compartments of oilseed rape in our study, whereas a previous study reported that it was specific for wheat and barley [30]. *Bacillus* species offer a multitude of plant-beneficial traits [61], which might be differentially selected for by either wheat or oilseed rape. Some host-specific bacterial microbiota patterns only prevail within a specific environmental context, such as edaphic conditions [13, 60], crop rotation [27], and nitrogen fertilization regime [62], while others are considered heritable [57]. Additionally, in our study we observed assignments of the same biomarker taxa to different crop species only differing by the two rhizosphere compartments We found an assignment of *Streptomycetaceae* as a biomarker of oilseed rape rhizosphere microbiome. On the other hand, they are observed to be recruited to the wheat root endosphere, but are consuming root exudate within the rhizospheres [60]. Hence, their plant host-specific recruitment might be restricted to the endosphere compartment, while they only traverse the rhizosphere microbiome. In conclusion, the enrichment of individual biomarker taxa to a specific plant-host was not apparent from the LEfSe biomarker analysis. Based on unexpected and contrasting findings of *LEfSe* biomarker assignment to a crop species between the close and distant rhizosphere and compared to previous studies, we argue that additional techniques such as network analyses are necessary to delineate non-transient host-specific signatures within the rhizosphere microbiome and to rule out artefacts.

### Keystone *Bacteria* of the rhizosphere core microbiota are differentially associated among crops species

A functional perspective on the bacterial microbiota assembly [63] in regard to plant-beneficial effects seems to be a promising route to elucidate rhizosphere bacterial microbiota assembly, which can be considered as a result of functional rather than purely phylogenetic selection [5, 63]. We hypothesized that the host signatures discriminating host-specific bacterial microbiota persisting in our study rely on hub and differential associations of *Bacteria* as inferred from co-occurrence networks analyses. Since the bacterial microbiota description was based on active *Bacteria*, the observed associations likely reflected functionally relevant patterns of bacterial microbiota interconnection and indirect trophic links with the plant host [32].

Differences between the structures of crop bacterial microbiota were caused by sub networks of hubs and their cohort partners. A singular hub pattern for oilseed rape was found in our study similar to a previous study [29]. Unlike this recent study [29], unclassified Chloroflexi of group *‘*KD4*-*96’ was the single hub of the oilseed rape bacterial microbiota in our study instead of *Pseudoarthrobacter*. Nevertheless, the direct cohort nodes found in our study overlap, namely unclassified Chloroflexi of group *‘*KD4*-*96’ itself, *Paenarthrobacter,* and nodes with potentially similar metabolic capabilities of ammonia oxidation (i.e. *Nitrosospira*) [29]. Since this cohort subnetwork appears to be reproducible across two studies, it can be considered as a functional trait specific of the oilseed rape microbiome. This assemblage may aid future research to further characterize and harness crop specific rhizosphere microbiota.

We identified *Massilia* as a network hub in cereal core microbiota with a high frequency to form distinct associations among host plants. The importance of *Massilia* for the wheat and barley rhizosphere core bacterial microbiota has only recently been recognized [16]. The recovery of *Massilia* as an active microbiome member over several growth stages in our study supports the classification by a previous study that it is stable member of a rhizosphere guild with the ability to upregulate carbohydrate-active enzymes in response to rhizodeposits [56]. Concerning a rye microbiota-specific differential association of *Massilia* with *Opitutus* was found, which are known as degraders of xylan [56]. Such specific differential associations might indicate distinct usage of plant-derived compounds between the crop host species. However, our observations conflict with *Massilia* being considered as a transient member being only present at early growth stages [62, 64].

We determined unclassified *Tepidisphaeraceae* of the group ‘WD2101 soil group’ as a hub of barley and the most frequent and differentially associated node, forming majorly negative associations. It most likely interacted with *Nocardiodedes* and uncultured *Beijerinckiaceae* ‘alphaI cluster’ within rye and wheat hosts or with *Skermanella* and other genera of the *Actinobacteria*, such as *Cellulomonas*, within oilseed rape and barley. This implies a competitive role within the bacterial microbiota. *Bacteria* similar to ‘WD21-01 soil group’ possess organelle-like micro compartments specialized to decomposing sugars from plant cell walls [65]. These organelles are considered to be a crucial genomic trait of plant host-specific and heritable bacterial microbiota [57]. This, supports our assumption that ‘WD21-01 soil group’ was most likely involved with degradation of plant cell wall components.

Consequently, the examination of hubs and their cohort partners provided reproducible information to establish a comprehensive understanding on how the rhizosphere microbiome assembly differs among crop species. These findings may guide culture-based physiological and single cell approaches to resolve the nature of their specific interactions.

### Differential associations of broadly-affiliated *Bacteria* facilitate understanding of their plant-host specific role

*Bradyrhizobium* was a biomarker taxon of wheat and oilseed in our study. This genus is considered to be enriched by other crops such as oilseed rape and to vary across soils, too [16, 58, 59, 66]. We found *Bradyrhizobium* to be significantly differentially associated when comparing microbial co-occurrence within wheat and oilseed rape rhizosphere, and thus suggesting different functionalities. Besides *Bradyrhizobium* species capable of symbiotic N_2_ fixation, a generalist role within the rhizosphere is implied by the unspecific transcriptomic response of *Bradyrhizobium* to rhizodeposits [56, 67]. Thus, we assume a distinct functional role of *Bradyrhizobium* within the wheat and oilseed rape microbiome.

*Nocardioides* belonged to the top five differential associated nodes and participated within the hub cohort of wheat and barley, representing a biomarker taxon of the latter. Thus, it was presumably key to the orchestration of both rhizosphere bacterial microbiota, but likely promoted different microbial interactions. This might have been its antifungal activities [42, 68].

Thus, differential associations were not only resolving the role of similar hub *Bacteria* in different host crops, they also explain ambiguous assignment of core biomarker taxa (LEfSe) to more than one crop species. Consequently, host-specific rhizosphere microbiota assembly is not restricted to individual bacterial genera and is determined by the interactions of keystone *Bacteria*.

## Conclusions

Previous studies based on DNA analyses that considered the relevance of differentially abundant taxa and co-occurrence networks inferred single bacterial taxa for the plant microbiome interaction [10, 15, 30]. Our study focused on the metabolically active fractions of the bacterial microbiota (i.e. SSU RNA pool) which is more relevant as a target of green biotechnology exploiting native microbiomes for crop production [5]. We specified the extent of host plant species and family effect on bacterial microbiota structure in the rhizosphere while including two rhizosphere compartments of four common crop species belonging to *Poaceae* and *Brassicaceae*. A significant effect of host species and plant growth stages on the active bacterial microbiota was observed.

Further, we identified co-occurrence network hubs and examined their cohort partners. We highlight that these sub-networks have a superior role in core microbiota assembly and promote divergence to specific crop rhizosphere microbiomes. Hubs considered as keystone *Bacteria* that also had a potential role in rhizosphere guilds were among others (a) *Massilia* in barley and wheat, and (b) unclassified *Chloroflexi* of group *‘*KD4*-*96’ in the oilseed rape bacterial microbiota. Differential associations between the co-occurrence networks of the core bacterial rhizosphere microbiota revealed decisive insights into their structural similarities and differences between crop species. Among them, the distinct association of ‘WD21-01 soil group’ with various actinobacterial genera. Thus, the study provides a blueprint of interdependent active keystone *Bacteria* that are capable to establish in the microbiomes of crop species over vegetative and reproductive growth stages. We conclude that instead of singularly enriched *Bacteria* their associations in sub-networks imposed the plant host-specific signatures within the bacterial rhizosphere microbiota. Considering theses assemblages, will be essential to future approaches that aim to modulate and harness native crop microbiomes.

## Supplementary Information

**Additional file 1**. Bacterial composition of the core microbiota.

## Data Availability

The datasets generated and analysed during the current study are available in the NCBI sequence database under accession PRJNA746551.
