# Supplementary material for: Crop host signatures reflected by co-association patterns of keystone Bacteria in the rhizosphere microbiota
Source: Environ Microbiome. 2021 Oct 12;16:18. doi: 10.1186/s40793-021-00387-w (PMC8513244; doi:10.1186/s40793-021-00387-w)
Supplement: Supplementary file 1 — Additional file 1. Bacterial composition of the core microbiota. [file 40793_2021_387_MOESM1_ESM.docx]

**Supplementary Table S1** Sampling dates per crop species and growth stage

| **crop species** | **growth stage** | **sampling date** | **days post planting** |
| --- | --- | --- | --- |
| barley | booting | 2019-06-21 | 31 |
| oilseed rape | booting | 2019-06-21 | 31 |
| rye | booting | 2019-06-21 | 31 |
| wheat | booting | 2019-06-21 | 31 |
| barley | flowering | 2019-07-24 | 64 |
| oilseed rape | flowering | 2019-07-12 | 52 |
| rye | flowering | 2019-07-26 | 66 |
| wheat | flowering | 2019-07-12 | 52 |
| barley | ripening | 2019-08-02 | 73 |
| oilseed rape | ripening | 2019-08-02 | 73 |
| rye | ripening | 2019-08-07 | 78 |
| wheat | ripening | 2019-08-01 | 72 |

**
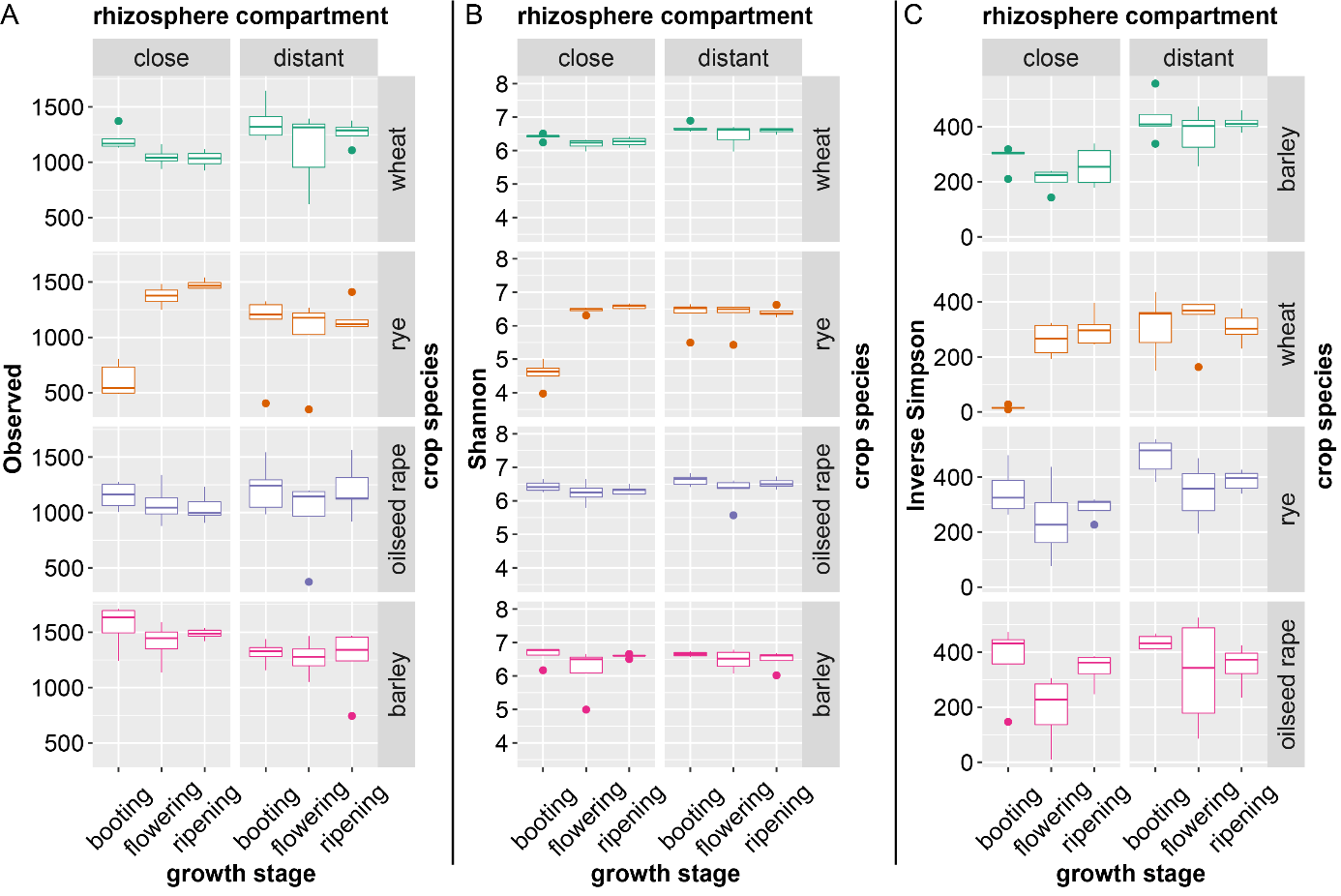
**

**Supplementary Figure 1** Alpha-diversity analyses of the close and distant bacterial rhizosphere microbiota: A: observed richness, **B:** Shannon index; **C:** inverse Simpson index**
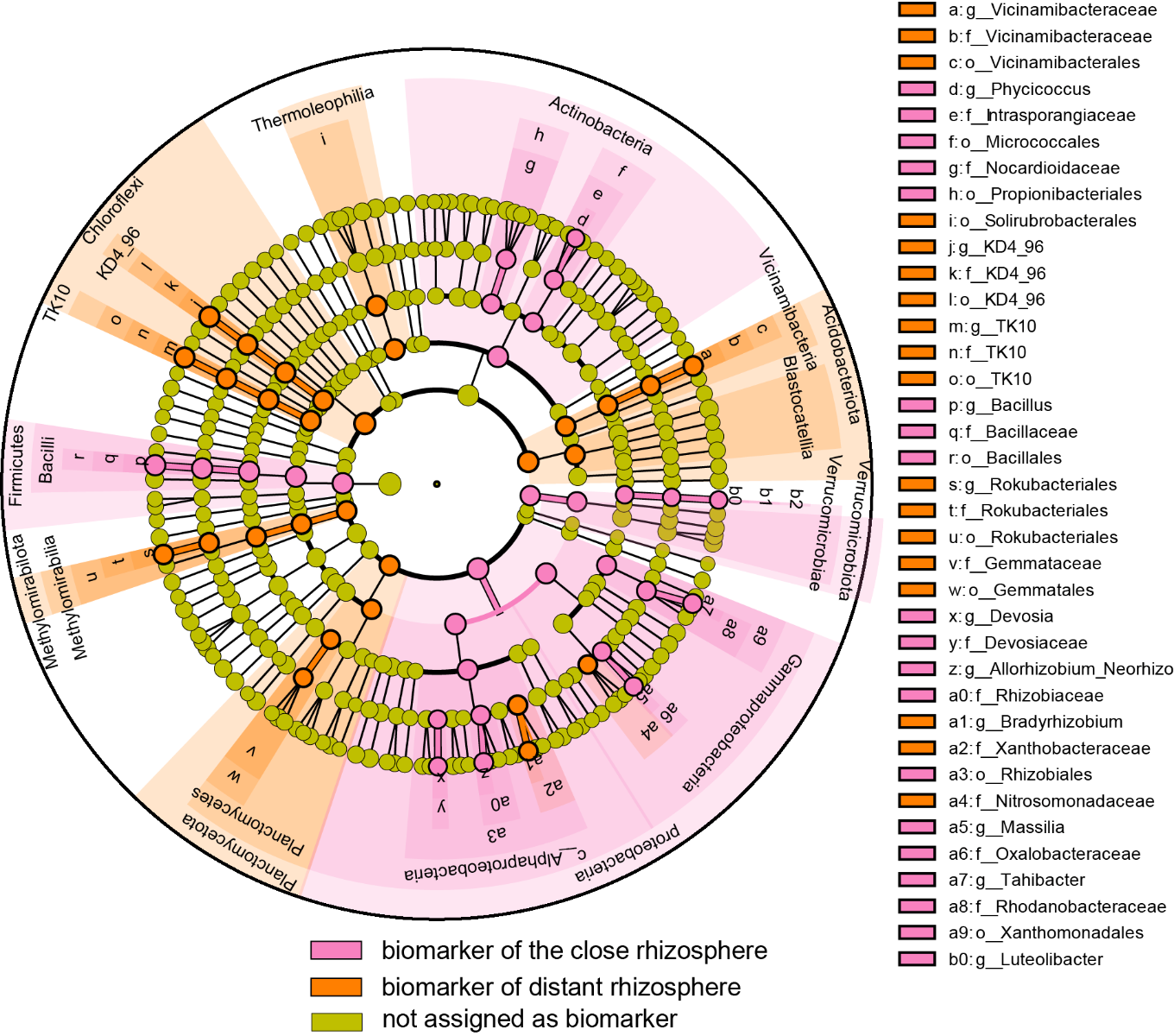
Supplementary Figure 2** Biomarker taxa indicative of the close and distant rhizosphere merged from the bacterial microbiota of wheat, barley, rye and oilseed rape identified by linear discriminant analysis effect size (LEfSe) visualized as circular taxonomic tree.

**
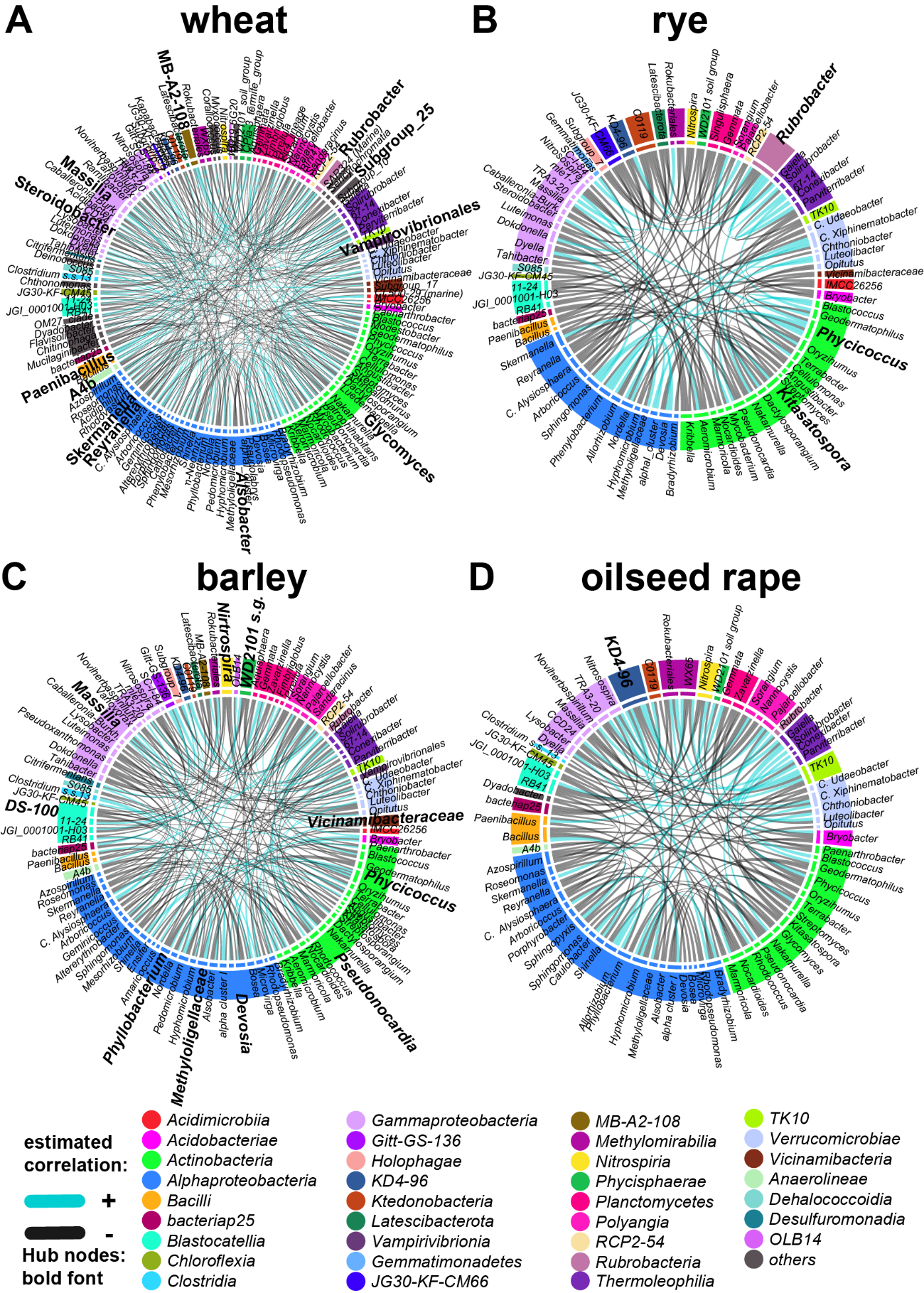
**

**Supplementary Figure 3** Co-association networks of the close rhizosphere bacterial core microbiota of wheat, rye, barley and oilseed rape (chord diagram) with hubs highlighted in bold font and sectors colored by taxonomic class.

**
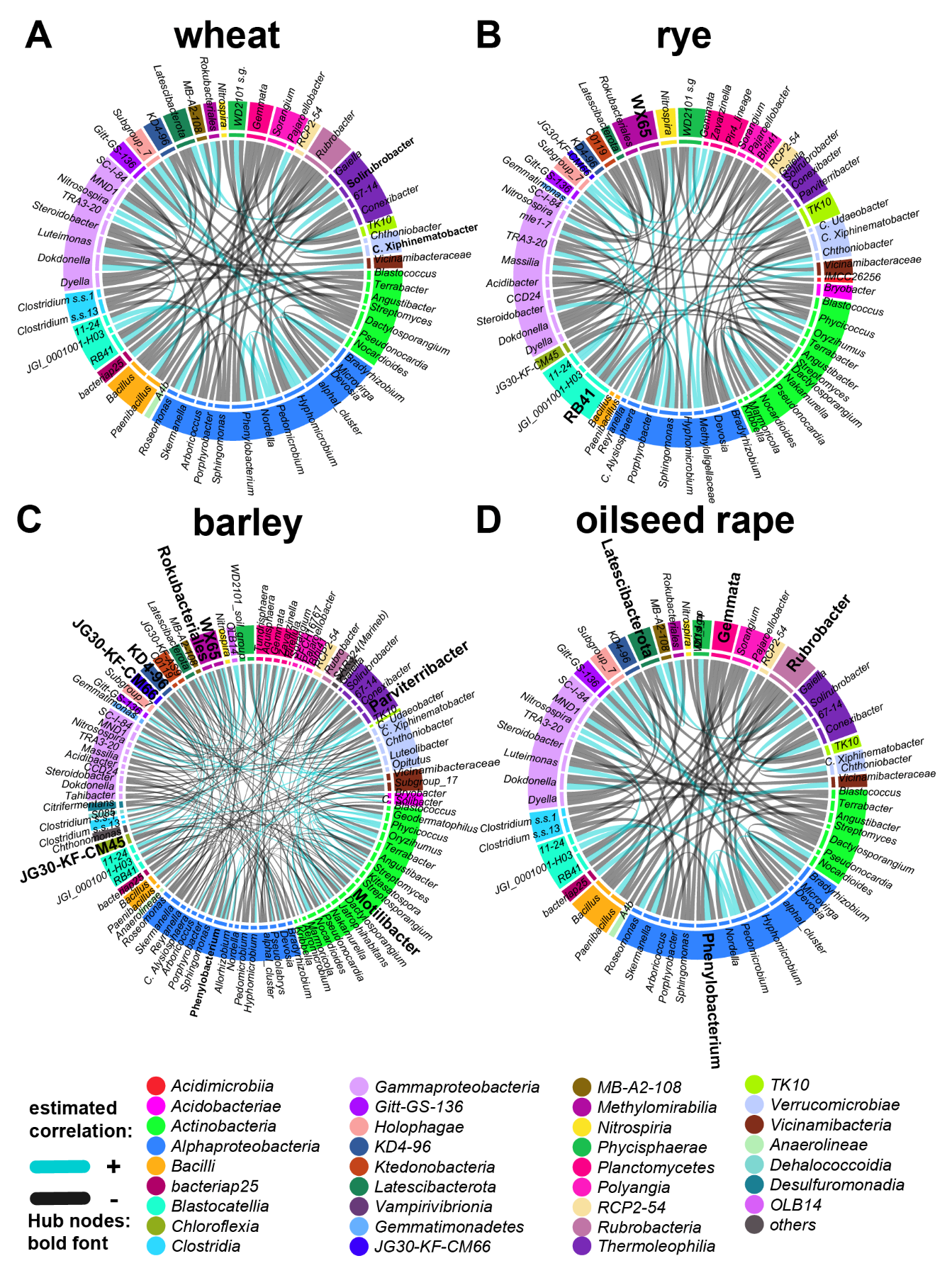
Supplementary Figure 4** Co-association networks of the distant rhizosphere bacterial core microbiota of wheat, rye, barley and oilseed rape (chord diagram) with hubs highlighted in bold font and sectors colored by taxonomic class.
